# Dexamethasone-induced cell death is restricted to specific molecular subgroups of multiple myeloma

**DOI:** 10.18632/oncotarget.4616

**Published:** 2015-07-16

**Authors:** Charlotte Kervoëlen, Emmanuelle Ménoret, Patricia Gomez-Bougie, Régis Bataille, Catherine Godon, Séverine Marionneau-Lambot, Philippe Moreau, Catherine Pellat-Deceunynck, Martine Amiot

**Affiliations:** ^1^ Myelomax, Nantes, France; ^2^ CRCNA, University of Nantes, INSERM, UMR892, CNRS, UMR 6299, Nantes, France; ^3^ Hematology Clinic, University Hospital, Nantes, France; ^4^ Hematology Laboratory, University Hospital, Nantes, France; ^5^ Plate-forme *in vivo*, Cancéropôle Grand Ouest, Nantes, France

**Keywords:** multiple myeloma, glucocorticoid, glucocorticoid receptor, GILZ, Bim

## Abstract

Due to its cytotoxic effect in lymphoid cells, dexamethasone is widely used in the treatment of multiple myeloma (MM). However, only a subset of myeloma patients responds to high-dose dexamethasone. Despite the undeniable anti-myeloma benefits of dexamethasone, significant adverse effects have been reported. We re-evaluate the anti-tumor effect of dexamethasone according to the molecular heterogeneity of MM. We demonstrated that the pro-death effect of dexamethasone is related to the genetic heterogeneity of MM because sensitive cell lines were restricted to *MAF* and *MMSET* signature subgroups, whereas all CCND1 cell lines (*n* = 10) were resistant to dexamethasone. We demonstrated that the glucocorticoid receptor expression was an important limiting factor for dexamethasone-induced cell death and we found a correlation between glucocorticoid receptor levels and the induction of glucocorticoid-induced leucine zipper (GILZ) under dexamethasone treatment. By silencing GILZ, we next demonstrated that GILZ is necessary for Dex induced apoptosis while triggering an imbalance between anti- and pro-apoptotic Bcl-2 proteins. Finally, the heterogeneity of the dexamethasone response was further confirmed *in vivo* using myeloma xenograft models. Our findings suggested that the effect of dexamethasone should be re-evaluated within molecular subgroups of myeloma patients to improve its efficacy and reduce its adverse effects.

## INTRODUCTION

Multiple myeloma (MM) is an incurable plasma cell malignancy despite considerable improvements to survival due to the introduction of new drugs, such as proteasome inhibitors and immunomodulatory agents [1]. Glucocorticoids (GCs) are widely used in the treatment of MM, mainly in combination regimens. Among GCs, dexamethasone (Dex) is used in all phases of treatment, including induction, consolidation and maintenance. In 1992, Alexanian *et al* showed that high doses of Dex were effective in about half of untreated MM patients and that the efficacy of the combination of vincristine, doxorubicin and dexamethasone was mainly due to the high doses of Dex [2]. Following the introduction of thalidomide, Dex has mainly been used in combination regimens because the combination of Dex with thalidomide demonstrates significantly superior response rates in newly diagnosed MM patients compared with Dex alone [3]. More recently, Dex associated with bortezomib and lenalidomide has appeared as the most promising drug association recommended for high-risk patients [4].

The effects of GCs occur through GC binding to the GC receptor GR, a member of the type I nuclear receptor superfamily. GR is transcribed from a single gene, *NR3C1*, located on chromosome 5. However, GR actions are dependent on multiple receptor isoforms. Alternative splicing of GR pre-mRNA produces five distinct GR protein isoforms: the predominant full-length GRα, the dominant negative GRβ, and the three less-well characterized and less abundant isoforms GRγ, GR-A and GR-P [5, 6]. Resistance to GCs is not fully understood because multiple molecular mechanisms are involved and are likely cell-type specific [7, 8]. However, altered or low-level GR expression accounts for inherent resistance to GCs [8]. Resistance may also occur downstream of GR. GCs bind GR, which then translocates to the nucleus and interacts with either GC-response elements to induce gene transcription (transactivation) or directly interacts with transcription factors, such as NFKB or AP-1 to repress their activity (transrepression). The transactivation activity of GR is highly regulated and required for GC-induced apoptosis involving the intrinsic mitochondrial pathway [6, 9]. The *TSC22D3* gene encoding the GC-induced leucine zipper protein GILZ is one of the most strongly up-regulated genes by GCs [10]. An important overlap between the effect of GILZ and those of GCs was demonstrated, suggesting that GILZ is a critical mediator of the therapeutic effect of GCs [11–13]. GILZ protein is involved in numerous protein/protein interactions and thus regulates multiple signaling pathways, including NF-κB and Ras [11–13].

Among the multiple candidate genes involved in GC-induced apoptosis, *BCL2L11*, which encodes the pro-apoptotic Bim protein, appears to be the most important. In 2003, *BCL2L11* was identified as a GC-induced death gene (18). Since that time, numerous studies have shown that Bim is a key mediator of GC-induced cell death in lymphoid cells [9, 14–16].

MM is molecularly heterogeneous, with chromosomal abnormalities that include full or partial deletions of chromosomes 13 or 17, amplification of 1q21, recurrent translocations of 14q32 or hyperdiploidy [17–19]. The recurrent 14q32 translocation and hyperdiploidy are associated with distinct gene expression profiles that define several groups, namely the HY, MS, CD-1/2 and MF groups, characterized by the hyperdiploid, *MMSET*, *CCND1* and *MAF* signatures, respectively. The MS and MF subgroups have been associated with poor overall survival [20, 21]. Although c-maf translocation is only found in 5 to 10% of patients, c-maf is overexpressed in approximately 50% of patients and is regulated both by the MEK and MMSET pathways [22, 23].

Because only 50% of untreated MM patients respond to high doses of Dex [2], our study was undertaken to re-evaluate the anti-tumor effect of Dex according to the molecular heterogeneity of MM patients using a large collection of myeloma cell lines (*n* = 31) that are representative of the molecular translocations found in patients.

## RESULTS

### The cellular response to Dex was correlated with the molecular subtype of human myeloma cell lines (HMCLs)

**Figure 1:**
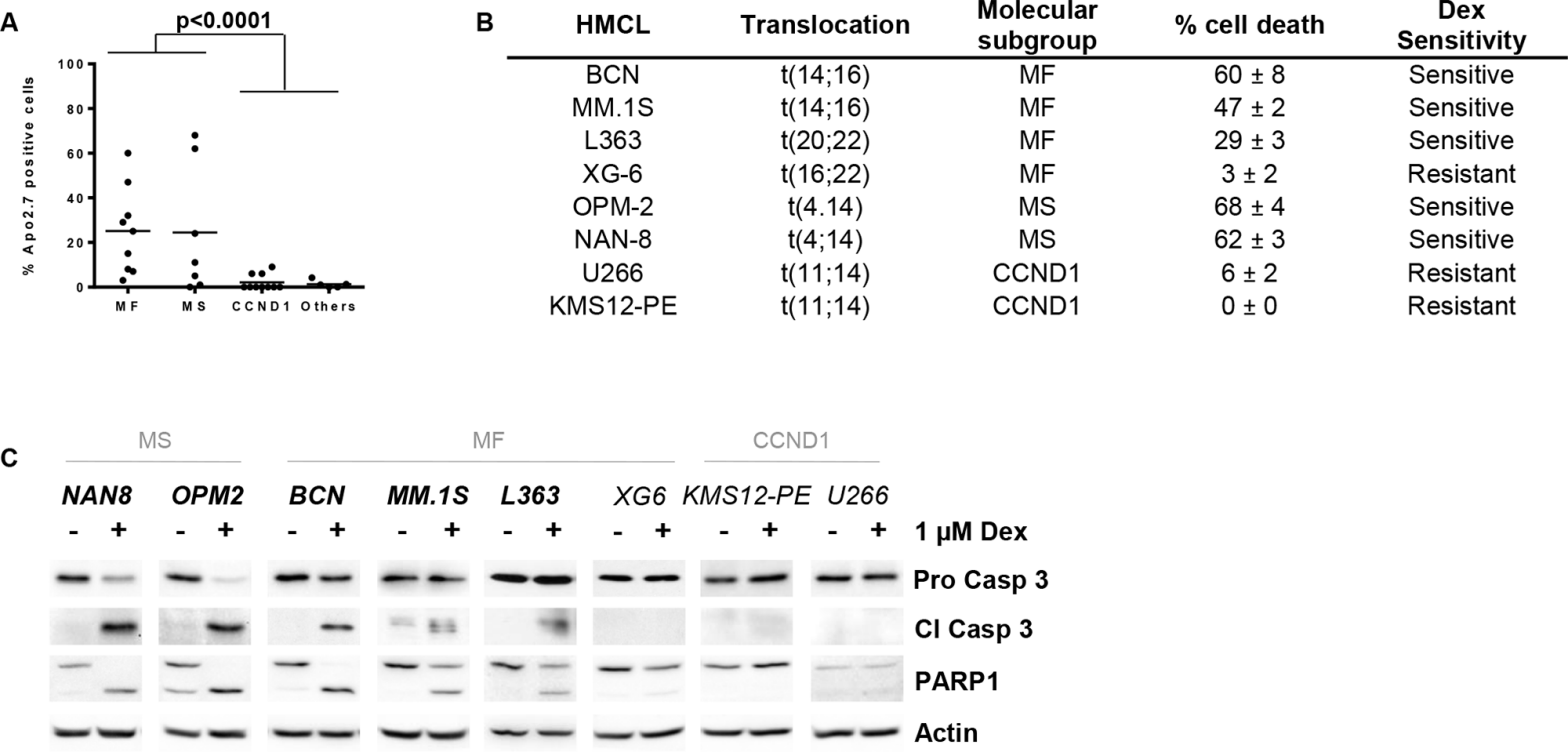
Dex sensitivity is heterogeneous and restricted to the MS and MF HMCL subgroups **A.** HMCLs (*n* = 31) were treated with 1 μM Dex for 72 hours, and cell death was assessed by Apo2.7 staining. HMCLs were classified into 4 groups as follow: MS, MF, CCND1 and Others for no or no recurrent translocation cell lines. Of note, HMCLs harboring a t(14;20), t(20;22) or t(16;22) were included in the MF subgroup. Each symbol represents one HMCL. Statistical analysis was performed using the Mann Whitney test. **B.** Characteristics of selected sensitive and resistant cell lines. Dex sensitivity was defined as follows: sensitive, ≥ 15% apoptotic HMCL cells and resistant, < 15% apoptotic HMCL cells. **C.** Cell death signature was analyzed by immunoblotting after 24 hours of Dex treatment. Actin was used as a loading control.

Although it is well accepted that Dex induces apoptosis in MM, the variability of the response among HMCLs with different genetic backgrounds harboring the main recurrent translocations has never been investigated. HMCLs were classified into MF, MS and CCND1 subgroups according to their IgH translocation (Supplementary Table S1). All HMCLs that did not present the main recurrent translocations leading to *MAF*, *MMSET* or *CCND1* over-expression were classified into the “Others” subgroup. Cell death induction was assessed across a panel of 31 HMCLs following Dex treatment for 72 hours. The dose of 1 μM of Dex was chosen because there is no dose response effect in cell death induction for Dex concentrations ranging from 0.1 to 10 μM (Supplementary Figure S1A). The ability of Dex to induce myeloma cell death was very heterogeneous within the different subgroups of HMCLs (Figure 1A, Supplementary Table S1). Dex-induced apoptosis was restricted to the MF and MS subgroups, with a mean number of apoptotic cells of 25% and 24%, respectively (Figure 1A). In contrast, no cell death was induced by Dex in any of the CCND1 HMCLs (*n* = 10). We first determined whether cell death was associated with caspase activation and PARP1 cleavage in a selected panel of cell lines, including Dex-sensitive (>15% cell death) and Dex-resistant HMCLs, which were representative of the molecular diversity of MM (Figure 1B). The results in Figure 1C showed that the activation of caspase 3 and the PARP1 cleavage were effective in all sensitive cell lines. Then, the effect of Dex was assessed in 19 primary myeloma cells, showing that cell death induction was highly heterogeneous from 0 to 96% (Table 1, Supplementary Figure S1B). Four samples harbored an IgH translation, one with a t(14;16), one with a t(4;14) and two with a t(11;14). Of note, the MF and MS patient were sensitive (92% and 49% cell death respectively), whereas the CCND1 patients were resistant (2% and 4% cell death). Two samples at diagnosis appeared resistant to Dex, indicating that primary tumor cells could present an inherent resistance to Dex.

**Table 1:** Dex sensitivity and characteristics of primary myeloma cells

Patient characteristics				Dex sensitivity	FISH analysis		
Sample	Disease	Status	Origin	% cell death (1 μM)	t (4;14)	t (14;16)	t (11;14)
1*	MM	R	BM	96	−	−	−
2	pPCL	D	PB	92	−	+	−
3*	MM	R	BM	87	−	−	−
4	pPCL	D	PB	84	−	−	−
5	MM	R	BM	60	−	−	−
6	MM	R	BM	58	−	−	−
7	MM	D	BM	49	+	−	−
8*	MM	D	BM	33	−	−	−
9	pPCL	D	PB	29	−	−	−
10	pPCL	D	PB	26	−	−	−
11	pPCL	D	PB	20	−	−	−
12	sPCL	R	PB	17	−	−	−
13*	MM	R	BM	8	−	−	−
14	MM	R	BM	8	−	−	−
15	MM	R	BM	4	−	−	+
16	sPCL	R	PB	2	−	−	+
17	sPCL	R	PB	0	−	−	−
18*	MM	D	BM	0	−	−	−
19	pPCL	D	PE	0	−	−	−

Abbreviations: MM, multiple myeloma; pPCL, primary plasma cell leukemia; sPCL, secondary plasma cell leukemia; D, diagnosis; R, relapse; BM, bone marrow; PB, peripheral blood; PE, pleural effusion.

Plasma cells were purified (*) or not with CD138-immunomagnetic beads depending on the myeloma infiltration and the total number of cells. Cell death was assessed after 48 hours Dex treatment by the loss of CD138 staining.

### High *NR3C1* expression characterized the MF and hyperdiploid (HY) subgroups of myeloma patients

**Figure 2:**
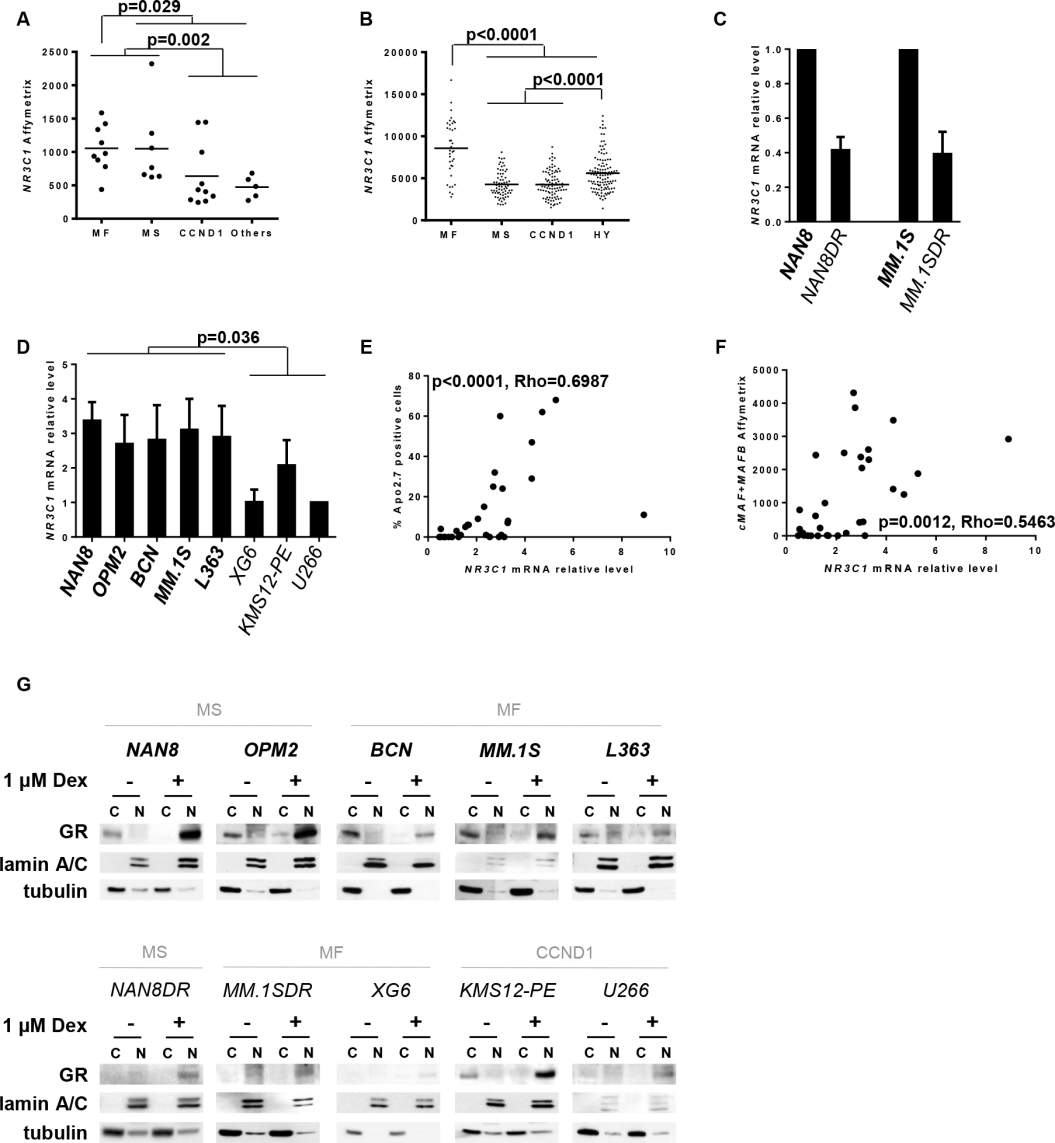
Analysis of both *NR3C1* gene and GR protein expression in myeloma subgroups **A, B.** *NR3C1* gene expression of HMCLs and 309 newly diagnosed MM patients was assessed by transcriptomic Affymetrix analysis and plotted for the respective HMCL subgroups (A), or for the MF, MS, CCND1 and hyperdiploid (HY) patient groups (B) Each symbol represents one HMCL or one patient. Statistical analysis was performed using the Mann Whitney test. **C, D.** *NR3C1* mRNA expression of Dex-resistant cell lines (C) and HMCL collection (D) was assessed by Q-PCR. *NR3C1* mRNA expression of generated Dex-resistant cell lines was normalized to *NR3C1* mRNA expression of the parental cell lines. *NR3C1* mRNA expression of HMCLs was normalized to *NR3C1* mRNA expression of U266 cell line. The mean ± SD of 3 experiments is presented. Sensitive cell lines are indicated by thick characters, and resistant cell lines are indicated by thin characters. Statistical analysis was performed using the Mann Whitney test. **E, F.** The relative *NR3C1* mRNA level was analyzed versus the percentage of Apo2.7-positive cells under Dex treatment (E) or versus *MAF* Affymetrix expression (F). *MAF* gene expression was assessed by addition of the *cMAF* and *MAFB* values of transcriptomic Affymetrix expression. Spearman's rank correlation coefficients are indicated. **G.** After 24 hours of Dex treatment, the translocation of GR was assessed by analyzing the expression of GR in both the nuclear (N) and cytosolic (C) fractions. Each fraction (20 μg) was subjected to western blot analysis for GR expression. Lamin A/C and tubulin were included as fraction purity markers. Sensitive cell lines are indicated as above.

Because Dex exerts its action through the GR, we investigated GR expression by transcriptomic Affymetrix analysis of the *NR3C1* gene in the different HMCL subgroups. *NR3C1* gene expression was heterogeneous among the HMCL subgroups (*p* = 0.02); however, the MF and MS subgroups exhibited a higher expression compared with the CCND1 and Others subgroups (1.8-fold mean increase, *p* = 0.002) (Figure 2A). We also found that *NR3C1* expression was significantly higher in the MF subgroup compared with all other HMCLs (1.4-fold mean increase, *p* = 0.029) (Figure 2A). We then took advantage of the publicly available Affymetrix gene expression microarray data from 309 newly diagnosed MM patients to analyze *NR3C1* expression in the MF, MS, CCND1 and HY groups of patients, as previously defined [18]. We first noticed that none of the patients completely lacked *NR3C1* expression but that *NR3C1* levels were significantly different between the molecular subgroups (*p* < 0.0001). Of note, MF patients expressed significantly higher levels of *NR3C1* than all other subgroups (1.8-fold mean change, *p* < 0.0001) (Figure 2B), suggesting that the specific regulation of *NR3C1* expression may occur in MF patients. Moreover, HY patients expressed significantly higher levels of *NR3C1* than MS and CCND1 patients (1.3-fold mean increase, *p* < 0.0001) (Figure 2B), which may be explained by the localization of the *NR3C1* gene on chromosome 5. Indeed, chromosome 5 trisomy is frequent in hyperdiploid MM [24].

### Levels of GR expression appear to be a limiting factor for Dex-induced cell death

To deepen the understanding of Dex sensitivity, we established two Dex-resistant cell lines, NAN8DR and MM.1SDR, obtained by long-term culture of the parental cell lines with a low Dex concentration (Supplementary Figure S2). NAN8DR and MM.1SDR cells were characterized by an absence of cell death induction (0% cell death in both NAN8DR and MM.1SDR compared with 58% and 35% in the parental cell lines, respectively) associated with nearly no caspase activation and no PARP1 cleavage under Dex treatment (Supplementary Figure S2). In contrast, their sensitivity to bortezomib or lenalidomide was unchanged (Supplementary Figure S2). To determine whether long-term Dex treatment affected the GR levels in resistant cell lines compared with their respective parental cell lines, we assessed *NR3C1* levels by Q-PCR. NAN8DR and MM.1SDR cells expressed very low *NR3C1* levels compared with the parental cell lines (Figure 2C). We next compared the *NR3C1* levels in our selected panel of cell lines and showed that sensitive cell lines demonstrated higher *NR3C1* levels than resistant cell lines (*p* = 0.036) (Figure 2D). However, we noticed that among the resistant cell lines, KMS12-PE expressed higher *NR3C1* levels than other resistant cell lines. Additionally, with some exceptions, we showed a correlation between *NR3C1* mRNA expression and the pro-apoptotic effect of Dex (Figure 2E, *p* < 0.0001, Rho: 0.70). Finally, we found a correlation between *NR3C1* and *MAF* expression suggesting that *NR3C1* expression may be linked with *MAF* expression (Figure 2F, *p* = 0.0012, Rho = 0.55). In its inactive form, GR is located in the cytoplasm; once activated by GC, it is translocated to the nucleus [25]. Thus, we analyzed GR protein expression in the cytosolic fraction and its translocation to the nucleus. Dex treatment induces the total translocation of GR to the nuclear fraction irrespective of its endogenous level (Figure 2G). Altogether, these results suggested that low levels of GR appear to be one of the first limiting events in Dex resistance.

### The strong up-regulation of GILZ was necessary for Dex-induced apoptosis

**Figure 3:**
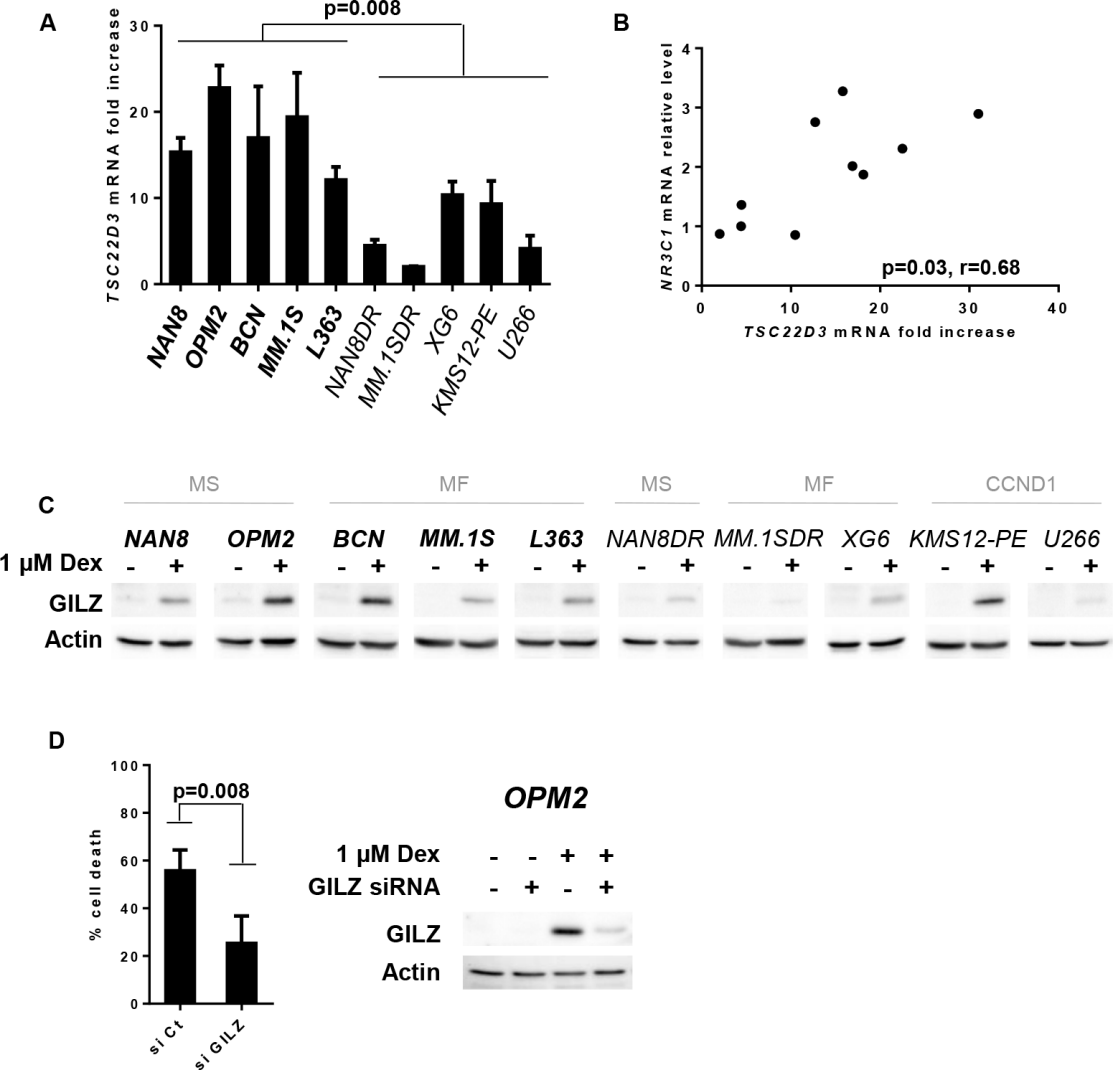
GILZ up-regulation is necessary for Dex-induced apoptosis **A.** *TSC22D3* mRNA expression was assessed by Q-PCR after 24 hours of Dex treatment. *TSC22D3* mRNA induction was assessed by calculating the fold increase relative to the untreated cell line. The mean ± SD of 3 experiments is presented. Statistical analysis was performed using the Mann Whitney test. **B.** Analysis of the relative *NR3C1* mRNA level versus the fold increase of *TSC22D3* mRNA under Dex treatment. Spearman's rank correlation coefficient is indicated. **C.** Cell lines were treated with Dex for 24 hours and cell lysates were immunoblotted with a GILZ antibody. **D.** Following GILZ silencing, OPM2 cells were treated with Dex. Cell death was assessed by Apo2.7 staining after 48 hours of Dex treatment. Data represent the mean ± SD of 4 independent experiments. Statistical analysis was performed using Wilcoxon matched-pairs signed rank test. After 16 hours of Dex treatment, GILZ silencing was analyzed by immunoblotting.

Using the same panel of HMCLs, we further investigated the expression of GILZ, a protein encoded by the *TSC22D3* GC-transactivated gene. After Dex exposure, *TSC22D3* levels were up-regulated in most cell lines (Figure 3A). However, resistant cell lines failed to up-regulate *TSC22D3* to the same extent as sensitive cell lines (Figure 3A). Of note, a correlation was found between the levels of GR expression and Dex-induced *TSC22D3* up-regulation (*p* = 0.03, Rho: 0.68) (Figure 3B). Analysis of the GILZ protein under Dex treatment confirmed a stronger up-regulation of GILZ not only in sensitive cell lines but also in KMS12-PE cells, which expressed high levels of GR (Figure 3C). To assess whether GILZ may influence the Dex response, we next performed transient knockdown of GILZ in both OPM2 and BCN cell lines. Indeed, GILZ silencing strongly reduced Dex-induced apoptosis (54% decrease, *p* = 0.008) in OPM2 cell line (Figure 3D) and to a lesser extent in BCN (30% decrease, *p* = 0.03) (Supplementary Figure S3). However, this weaker effect on Dex-induced apoptosis in BCN compared to OPM2 is probably due to the less efficient GILZ silencing (40% versus 83% inhibition respectively). Altogether, these results highlighted the implication of GILZ in Dex-induced apoptosis.

### GILZ was involved in Bim up-regulation and Bcl-x_L_ down-regulation associated with Dex-induced apoptosis in HMCLs

**Figure 4:**
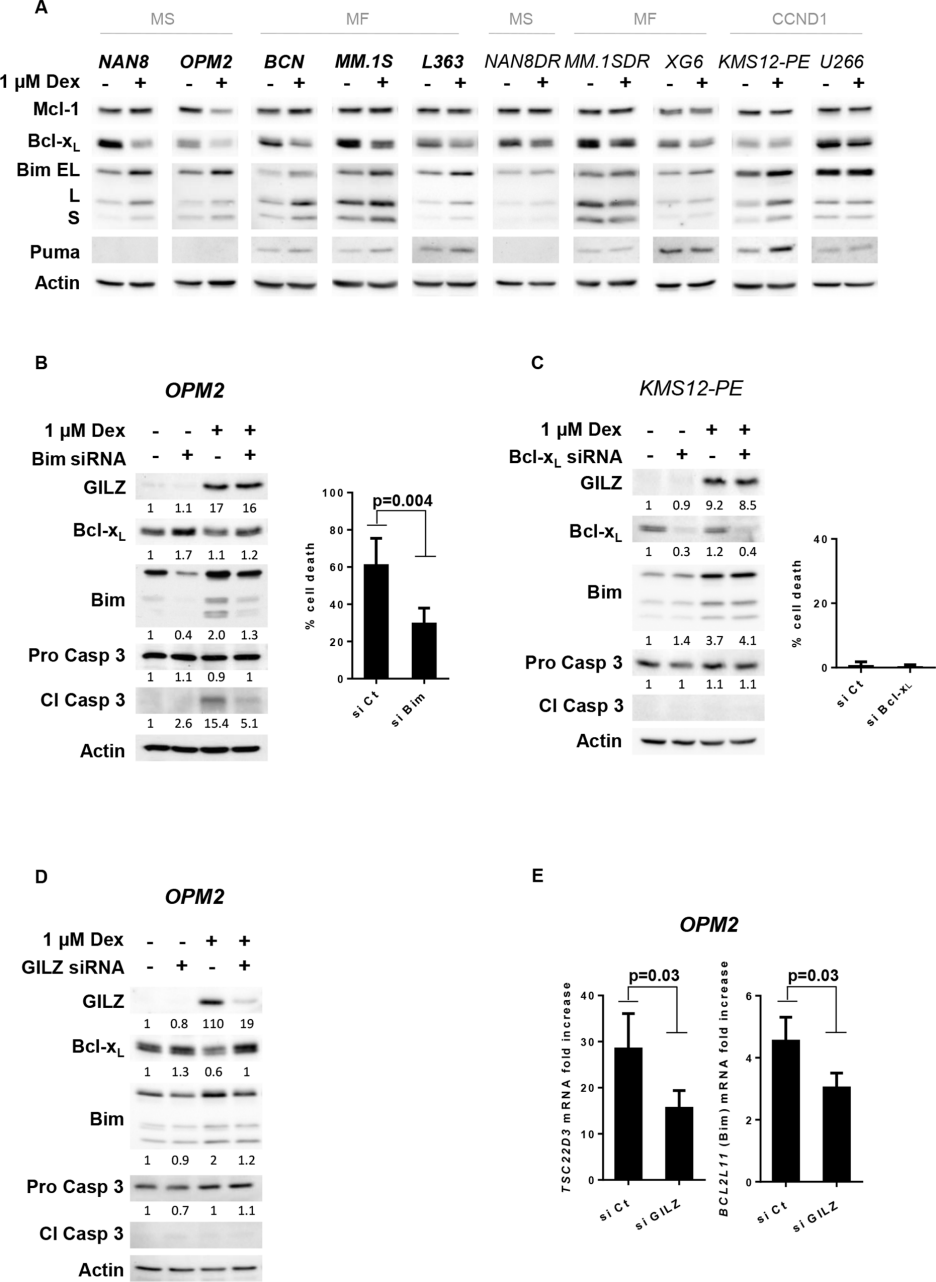
GILZ is involved in Bim up-regulation and Bcl-x_L_ down-regulation associated with Dex-induced apoptosis **A.** Cell lines were treated with Dex for 24 hours and lysates were immunoblotted with Mcl-1, Bcl-x_L_, Bim and Puma antibodies. **B.** Following siRNA transfection, OPM2 cells were treated with Dex for 48 hours. The expression of Bim, Bcl-x_L_, GILZ and caspase 3 were analyzed by immunoblotting and normalized (arbitrary unit) to actin expression. Apoptosis was quantified by Apo2.7 staining. The results show the mean ± SD of 4 independent experiments. Statistical analysis was performed using Wilcoxon matched-pairs signed rank test. **C.** Following siRNA transfection, KMS12-PE cells were treated with Dex for 48 hours. The protein expressions were analyzed by immunoblotting and normalized (arbitrary unit) using actin expression. Apoptosis was quantified by Apo2.7 staining. The results show the mean ± SD of 3 independent experiments. **D.** GILZ was transiently silenced in OPM2 cells for 48 hours, before to be treated with Dex for 16 hours. The protein expressions were analyzed by immunoblotting and normalized (arbitrary unit) to actin expression. **E.** Following GILZ silencing, OPM2 cells were treated with Dex for 8 hours. *TSC22D3* and *BCL2L11* mRNA induction was assessed by calculating the fold increase relative to the untreated cell line. The mean ± SD of 5 independent experiments is presented. Statistical analysis was performed using Wilcoxon matched-pairs signed rank test.

Among the pro-apoptotic Bcl-2 family proteins, Bim, a BH3-only member, has frequently been identified as one of the key mediators of GC-induced apoptosis [14–16]. In addition to Bim, Puma another BH3-only member was also shown to contribute to GC-induced apoptosis [15]. Furthermore, based on a previous microarray analysis in MM.1S cells [26], the *BCL2L1* gene that encodes Bcl-x_L_ was shown to be the only anti-apoptotic gene strongly down-regulated by Dex. We demonstrated that a strong up-regulation of all Bim isoforms occurred mainly in sensitive cell lines but also in KMS12-PE Dex-resistant cells (Figure 4A). When Puma was endogenously expressed in the HMCLs, we observed a weak Puma up-regulation in parallel to Bim up-regulation (Figure 4A). The anti-apoptotic Mcl-1 protein was not modulated with the exception of OPM2 where Mcl-1 is down-regulated. In contrast to Mcl-1, we showed that Bcl-x_L_ protein expression was strongly decreased in all Dex-sensitive HMCLs, whereas its expression was weakly modified in Dex-resistant HMCLs (Figure 4A). These results may indicate that the apoptotic response is associated with an imbalance between pro- and anti-apoptotic proteins. We next demonstrated that Bim silencing in OPM2 cells caused a significant decrease in Dex-induced cell death (51%, *p* = 0.004) (Figure 4B). Finally, because the resistant KMS12-PE HMCL is characterized by an increase in Bim under Dex treatment but an absence of Bcl-x_L_ down-regulation, we silenced Bcl-x_L_ to determine whether its down-regulation could overcome Dex resistance. Despite the complete silencing of Bcl-x_L_ in KMS12-PE cells, Dex did not induce significant cell death, indicating that Bcl-x_L_ down-regulation concomitant to Bim up-regulation was not sufficient to counteract Dex resistance in this CCND1 HMCL (Figure 4C). Of interest, the silencing of GILZ impairs both Bim up-regulation and Bcl-x_L_ down-regulation under Dex treatment (Figure 4D). Finally, we demonstrated that GILZ silencing decreased *BCL2L11* (Bim) up-regulation at the transcriptional level (33% decrease, *p* = 0.03) (Figure 4E). Altogether, these results seem to indicate a pivotal role for GILZ in Dex-induced cell death through the regulation of the Bcl-2 protein network.

### Dexamethasone reduced tumor cell growth of *in vitro* sensitive but not resistant cells

**Figure 5:**
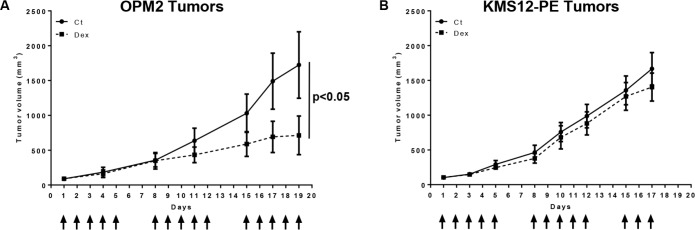
Dexamethasone reduces tumor cell growth of *in vitro* sensitive OPM2 but not resistant KMS12-PE cells in xenograft models **A.** Dex treatment inhibited *in vivo* OPM2 tumor growth. Mice were subcutaneously injected with 12 × 10^6^ OPM2 cells. The mice received either vehicle (Ct) or Dex (1 mg/kg, i.p.) 5 days per week for 3 consecutive weeks. Arrows indicate injections. The mice were then sacrificed on day 19. Statistical analysis was performed using a two-way ANOVA test, followed by a Bonferroni post-test. **B.** Dex treatment did not affect *in vivo* KMS12-PE tumor growth. Mice were subcutaneously injected with 12 × 10^6^ KMS12-PE cells and treated as above. The mice were then sacrificed on day 17. Statistical analysis was performed using a two-way ANOVA test, followed by a Bonferroni post-test.

To assess whether the differential *in vitro* activity of Dex could reflect its *in vivo* activity, both Dex-sensitive (OPM2) and Dex-resistant (KMS12-PE) cell lines were xenografted into SCID mice. Mice were subcutaneously inoculated with 12 × 10^6^ cells. When mice bore tumors of similar sizes, they were randomly separated into 2 groups and received either vehicle (control) or Dex (1 mg/kg) 5 days a week for 3 consecutive weeks (Figure 5). When the tumor sizes exceeded 2000 mm^3^, mice were sacrificed on day 19 and 17 for mice grafted with OPM2 and KMS12-PE cells respectively. Dex significantly inhibited OPM2 tumor growth (Figure 5A) (*p* < 0.05), but failed to reduce tumor growth of the *in vitro* resistant KMS12-PE cells (Figure 5B). Taken together, these data demonstrated that the *in vivo* pro-apoptotic Dex effect in pre-clinical myeloma models is related to *in vitro* Dex-induced cell death.

## DISCUSSION

Because clinical practice has frequently been based on empirical studies, our study was undertaken to re-evaluate Dex, a conventional therapy, according to the molecular classification of MM patients. Our results highlighted that the Dex pro-apoptotic effect was restricted to the MF and MS subgroups of HMCLs, whereas all other cell lines were totally resistant to Dex. In particular, all CCND1 HMCLs and primary samples were resistant to Dex-induced cell death. The analysis of Dex sensitivity in relation to *NR3C1* levels revealed that the MF subgroup expressed the highest levels of *NR3C1*, which may partially explain their stronger sensitivity to Dex. A previous study has also reported a link between GC and MAF showing that GC reduced c-maf protein levels via an ubiquitination-dependent degradation [27]. Nevertheless, more investigations will be necessary to explain the over-expression of *NR3C1* in the MF subgroup. In addition, the localization of the *NR3C1* gene on chromosome 5 probably explains the higher levels of *NR3C1* found in the HY subgroup compared with the MS and CCND1 subgroups because chromosome 5 trisomy is frequently found in hyperdiploid MM [24]. The generation of MF or MS Dex-resistant HMCLs after long-term exposure to Dex led to an important decrease in *NR3C1*, indicating that an *NR3C1* level seems necessary for Dex-induced apoptosis. To support this hypothesis, we showed a correlation between *NR3C1* levels and the apoptotic response in HMCLs. Consistently, a poor prognosis has been associated with low *NR3C1* expression in MM patients [28, 29]. In addition, we demonstrated that Dex induced the total translocation of GR to the nucleus in all HMCLs tested, even in resistant cell lines, showing that the ability of GR to translocate to the nucleus is not a limiting step in Dex resistance.

Many studies in hematopoietic cells, essentially in T and dendritic cells, have identified GILZ as one of the main GC-regulated genes [30, 31]. The GILZ promoter includes 6 GC-responsive element motifs that allow for GR homodimer binding and transactivation [32]. In the present study, we demonstrated that Dex induced an up-regulation of GILZ in all HMCLs but that this up-regulation was significantly higher in sensitive cell lines. Furthermore, we found a correlation between *NR3C1* levels and *TSC22D3* mRNA up-regulation. This result reinforces the fact that GILZ expression was proposed as a reliable measure of GR function [33]. We also demonstrated that GILZ silencing induced a strong reduction in Dex-induced cell death. This is in agreement with a study from Grugan *et al* [34], which has already implicated GILZ in Dex-induced apoptosis. However, in this previous study, the reduction in Dex-induced cell death caused by GILZ silencing was very modest (10%). Altogether, these data now clearly indicate a pivotal role of GILZ in the induction of cell death by Dex. Although Dex-induced apoptosis required the contribution of multiple processes, modification of the Bcl-2 protein rheostat has been reported as a key process [35]. Particularly, BH3-only members have been shown to be important mediators of GC-induced apoptosis [14–16]. In our HMCL collection, we demonstrated that a strong Bim up-regulation associated with a weak Puma up-regulation occurs mainly in Dex-sensitive cell lines, but also in the Dex-resistant KMS12-PE cell line. We further demonstrated that Bim silencing led to a strong reduction in apoptosis, which confirms the important role of Bim in the cell death process induced by Dex. In parallel with Bim up-regulation, we observed a strong down-regulation of Bcl-x_L_, which is restricted to sensitive HMCLs. Thus, an imbalance between anti- and pro-apoptotic molecules occurred, leading to a decrease in the apoptotic threshold in Dex-sensitive cell lines. Of interest, GILZ silencing provided evidence that GILZ contributed to the regulation of the Bcl-2 protein network by inducing Bim up-regulation and Bcl-x_L_ down-regulation. The regulation of Bcl-x_L_ by GILZ is in agreement with a previous study showing that Bcl-x_L_ is decreased in GILZ-overexpressing transgenic mice [36]. The implication of GILZ in Bim up-regulation remains an important question that deserves further investigation. Altogether, our data reinforce the importance of GILZ in the pro-apoptotic effect of GC in hematopoietic tumor cells.

Whereas CCND1 HMCLs have a low level of GR, which could in part account for their resistance to Dex, KMS12-PE cells exhibited a high level of GR, leading to both a significant induction of GILZ and up-regulation of Bim without the induction of apoptosis. Because Dex did not induced Bcl-x_L_ down-regulation in KMS12-PE cells as it did in other sensitive cell lines, we forced this down-regulation, showing that concomitant Bcl-x_L_ down-regulation, Bim up-regulation and GILZ induction were not sufficient to counteract the Dex resistance of KMS12-PE cells. We cannot rule out other factors that may contribute to the Dex response. Finally, the *in vivo* growth of myeloma cells in our mouse model confirmed the heterogeneity of Dex efficacy, which corroborated our *in vitro* findings. Indeed, whereas Dex led to a reduction of MS tumor growth, Dex had no effect on CCND1 tumor growth. Of note, the dual action of GCs in survival has been reported [37, 38]. Indeed, it was demonstrated that although Dex induced the apoptosis of a large majority of primary leukemia cells from children with newly diagnosed acute leukemia, the survival of a subset of primary acute leukemia samples (32%) was increased by GCs [37]. Furthermore, our results seem to indicate that Dex had no pro-apoptotic effects on CCND1 myeloma cells both *in vitro* and *in vivo*, despite the presence of effective GR. Interestingly, the CCND1 subgroup has already been distinguished from other subgroups by its particular anti- and pro-apoptotic Bcl-2 member expression and its specific drug response [39, 40]. Altogether, these results indicated that both the MF and CCND1 subgroups presented important specificities that require better analysis during the course of the disease and the response to treatment. The risk/benefit of prolonged GC treatment should be re-evaluated to propose optimal treatments by GCs for each molecular group of MM.

## MATERIALS AND METHODS

### Cell lines, primary myeloma cells and culture conditions

HMCLs BCN, MDN, SBN, NAN-1, -6, -7, -8, -10 and XG-1, -2, -3, -5, -6, -7 were derived in our laboratory from primary myeloma cells in the presence of interleukin (IL)-6. ANBL-6 was kindly provided by Dr. Jelinek (Rochester, MN). KMS11, KMS12-BM, KMS12-PE, and KMM1 were kindly provided by Dr. Otsuki (Kurashiki, Japan). JJN3, JIM3, Karpas620, and MM.1S were, respectively, kindly provided by Drs. Van Riet (Brussels, Belgium), MacLennan (Birmingham, UK), Karpas (Cambridge, UK), and S. Rosen (Chicago, IL). AMO1, LP1, L363, NCI-H929, SKMM2, U266, and OPM2 were purchased from the DSMZ, and RPMI8226 from American Type Culture Collection. Each HMCL was characterized and identified as previously described [41–43].

After obtaining informed consent, blood or bone marrow samples from MM patients were collected at the Department of Hematology at the University Hospital of Nantes or at the Intergroupe Francophone du Myélome (ethical approval n° DC-2011–1399, Pr Rodat). Plasma cells were purified with CD138 immunomagnetic beads. The purity of the plasma cells was greater than 90%, as assessed morphologically or by CD138 staining.

### Reagents

Dexamethasone was purchased from Sigma-Aldrich (Saint-Quentin Fallavier, France) and dissolved in ethanol at 1 mM as a stock solution. For the *in vivo* study, Dex was dissolved in ethanol and then in PBS at a final Dex concentration of 0.2 mg/ml, with a final ethanol concentration of less than 3%.

### Cell death assays

The evaluation of cell death in HMCLs was performed by flow cytometry using Apo2.7 staining. Cell death in primary myeloma cells was measured after 48 hours of culture in RPMI-1640 containing 5% FCS with or without Dex (1 μM) by assessing the loss of CD138 staining, as previously described [42].

### Establishment of Dex-resistant cell lines

To establish Dex-resistant cell lines, NAN8 and MM.1S cells were continuously treated with Dex beginning with a dose of 0.001 μM. After cells displayed a viability of 80% under continuous exposure to a low Dex concentration, the drug dose was doubled. This process was repeated until cells were cultured in a Dex concentration of 10 μM. Prior to the experiments, the cells were cultured in Dex-free medium for 5 days before use.

### Immunoblotting

Western blotting analyses were performed as previously described [44]. The following antibodies were used: caspase-3 (E-8), GR (E-20), lamin A/C (E-1), GILZ (FL-134), Mcl-1 (S-19) (Santa Cruz, Heidelberg, Germany), Puma (D30C10) (Cell Signaling Technology, Saint Quentin, France), tubulin (GE Healthcare Life Sciences, Velizy, France), Bcl-x_L_ (BD Transduction Laboratories, Rungis, France), Bim, PARP1 (Ab-2) and actin (Merck Chemicals, Nottingham, UK). Protein expression was quantified and normalized to actin expression using ImageJ software.

### RNA interference assays

Control nontargeted siRNA, siBim and siGILZ were purchased from Santa Cruz Biotechnology (Heidelberg, Germany), and siBcl-x_L_ was purchased from Thermo Scientific (Ilkirch, France). OPM2 cells were transfected using Lipofectamine RNAiMAX (Invitrogen, Life Technologies, St Aubin, France) according to the manufacturer's instructions. Briefly, cells were plated at 1 × 10^6^ cells per well in a 6-well plate. After 24 hours, 100 pmol siRNA was transfected into the cells using Lipofectamine RNAiMAX reagent. Cells were incubated for 72 hours for siBim and 48 hours for siGILZ before being subjected to various analyses. KMS12-PE and BCN cells were transfected using the Amaxa 4D-Nucleofector (Lonza, Cologne, Germany). The cell pellet was resuspended with 100 μl solution F (Lonza) containing 100 pmol siRNA and electroporated using the CA137 or the DN100 program for KMS12-PE and BCN, respectively. After 24 hours, the cells were treated with Dex.

### Affymetrix gene expression

Gene expression analyses of newly diagnosed MM patients were performed with the publicly available Affymetrix Amazonia database (http://amazonia.transcriptome.eu/). HMCL gene expression analyses were performed with the RAGE database. The probes used for analyses were as follows: 211671_s_at for the *NR3C1* gene, 209348_s_at for the *cMAF* gene and 218559_s_at for the *MAFB* gene.

### RNA extraction, reverse transcription and quantitative real-time PCR

RNA extraction, reverse transcription and Q-PCR were performed as previously described [45]. TaqMan gene expression assays for *NR3C1* (Hs00353740_m1), *TSC22D3* (Hs00608272_m1), *BCL2L11* (Hs00708019_s1) and *RPL37A* (Hs01102345_m1, housekeeping gene) were obtained from Applied Biosystems (Life Technologies, St Aubin, France).

### Cell fractionation

Cell fractionation was performed using a Nuclear Extraction kit (Cayman Chemical, Ann Arbor, USA) by following the manufacturer's protocol. Briefly, the cytosolic fraction was first obtained after lysis using a hypotonic buffer. Then, the nuclear fraction was obtained after 30 minutes incubation with a nuclear extraction buffer.

### Xenotransplant

Female, beige, 6-week-old SCID mice were purchased from Charles River (L'Arbresle, France). Mice were bred and housed in the Experimental Therapeutic Unit (UTE, SFR Bonamy, Nantes, France) under animal care license n°44565. OPM2 or KMS12-PE tumors were generated by implanting 12 × 10^6^ cells in 100 μl of PBS/Matrigel (1:1, Corning) in the right flank above the hind leg [46]. The tumor volume was recorded in three dimensions using a digital caliper and calculated as the length × width × depth. When the mice bore similar tumor sizes (approximately 100 mm^3^), they were randomly separated into 2 groups (6 mice per group) and received an intraperitoneal injection (i.p.) of either vehicle (PBS-diluted ethanol) or Dex (1 mg/kg) 5 days a week for 3 consecutive weeks. Animals were sacrificed when their tumor sizes reached 2000 mm^3^.

### Statistical analysis

Statistical analyses were conducted using Mann–Whitney, Kruskal-Wallis, Spearman, Wilcoxon matched-pairs signed rank or a two-way ANOVA test.

## SUPPLEMENTARY FIGURES AND TABLE


