# Supplementary material for: Dexamethasone-induced cell death is restricted to specific molecular subgroups of multiple myeloma
Source: Oncotarget. 2015 Jul 16;6(29):26922–34. doi: 10.18632/oncotarget.4616 (PMC4694963; doi:10.18632/oncotarget.4616)
Supplement: Supplementary file 1 [file oncotarget-06-26922-s001.pdf]

SUPPLEMENTARY FIGURES AND TABLE

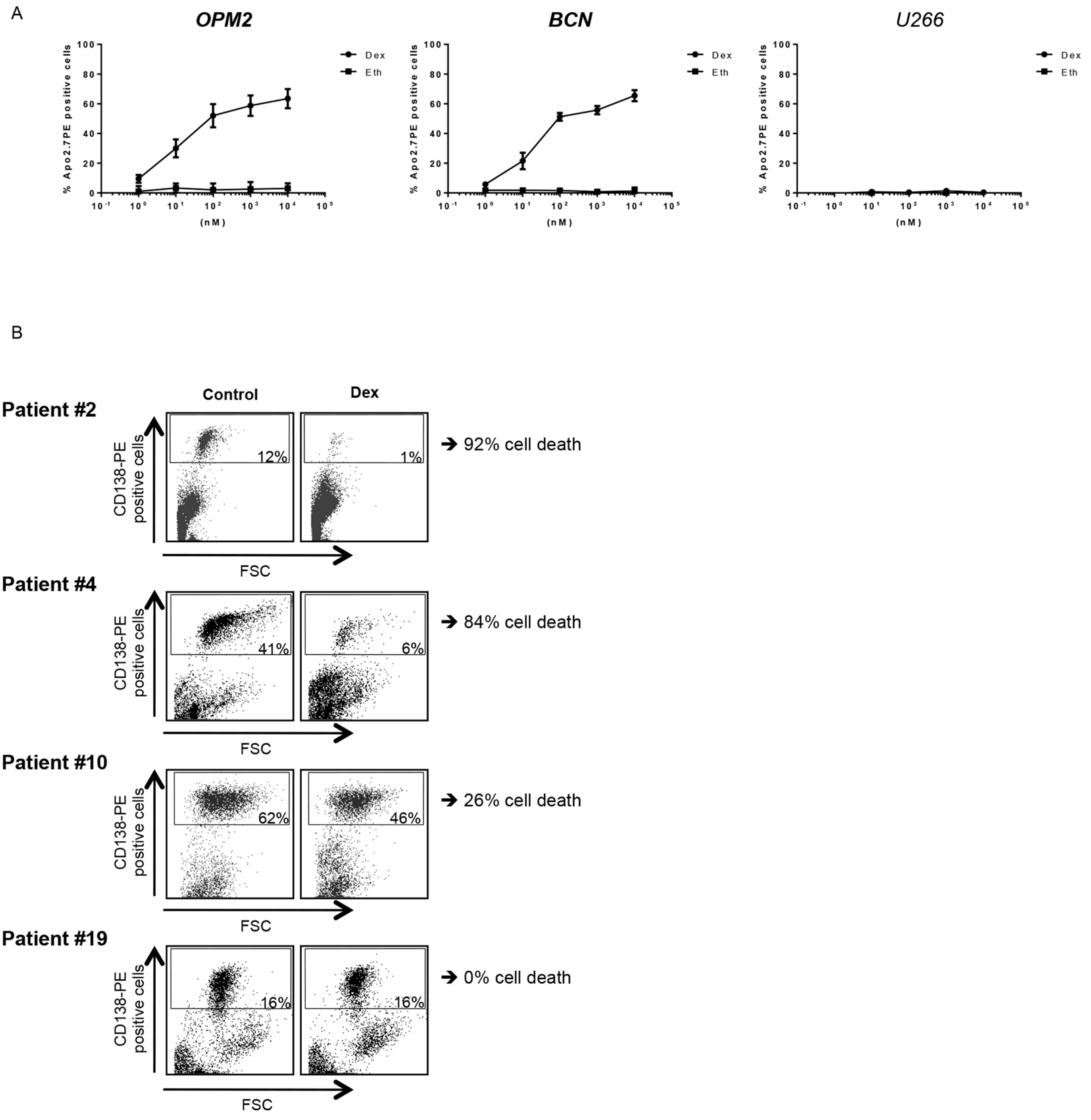

Supplementary Figure S1: A. OPM2, BCN and U266 cell lines were treated with Dex concentrations ranging from 1 to 10^4 nM Dex for 72 hours and cell death was assessed by Apo2.7 staining. B. Dex sensitivity of patient samples was assessed by measure of the loss of CD138 expression after 48 hours of 1 µM Dex.

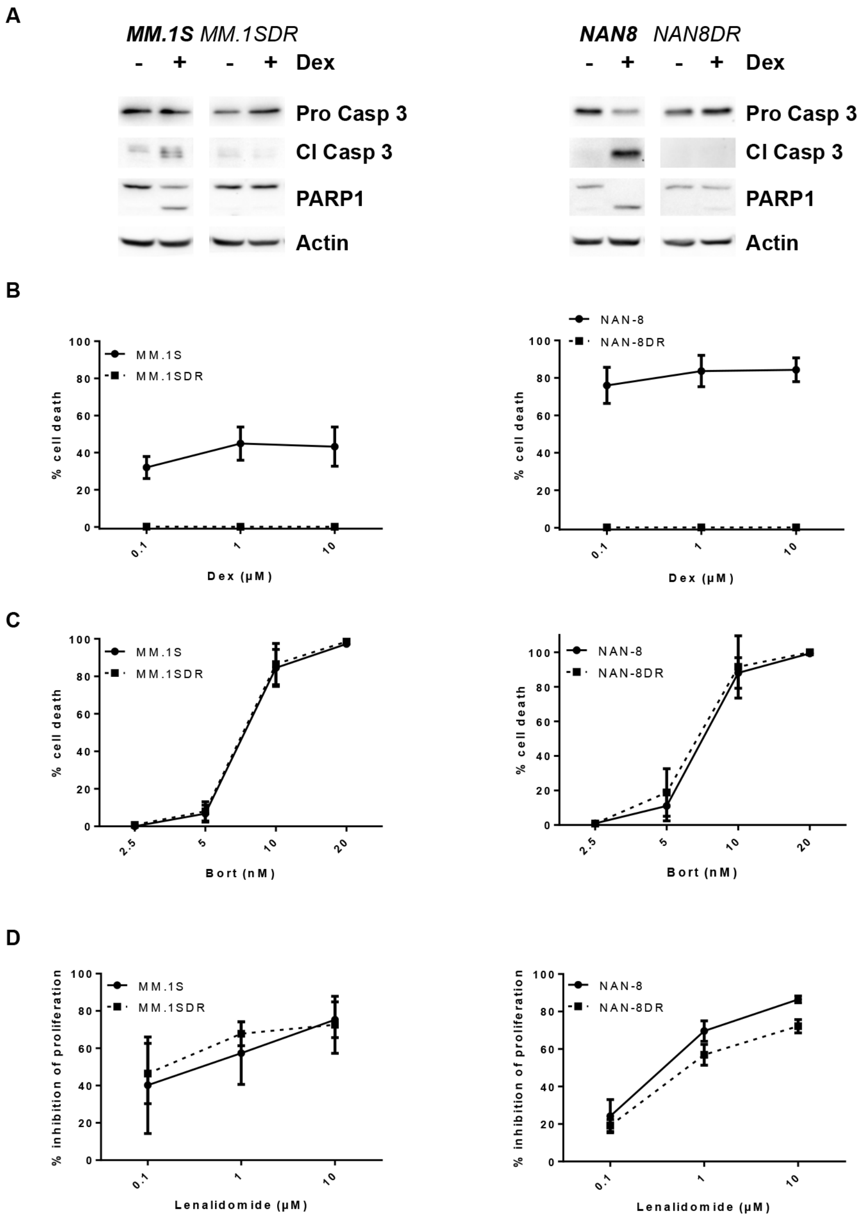

Supplementary Figure S2: A. Cell death signature was analyzed by immunoblotting after 24 hours of Dex treatment. Actin was used as a loading control. **B.** Dex-induced cell death was assessed by Apo2.7 staining after 72 hours of treatment with 0.1, 1 or 10 µM of Dex. Data represent the mean (±SD) of 4 experiments. **C.** Bortezomib induced cell death were assessed by Apo2.7 staining after 48 hours of treatment. Data represent the mean (±SD) of 4 experiments. **D.** Inhibition of proliferation induced by 72 hours of Lenalidomide treatment was assessed by measurement of incorporation of [^{3}H] Thymidine. Myeloma cells (10^{4} cells/well) were cultured in triplicate in 96-well plates for 72 hours. Cells were pulsed with 1 µCi [^{3}H]thymidine for the last eight hours of culture. The uptake of [^{3}H]thymidine was monitored using a 1450-Microbeta Jet beta-counter. Data represent the mean (±SD) of 4 experiments.

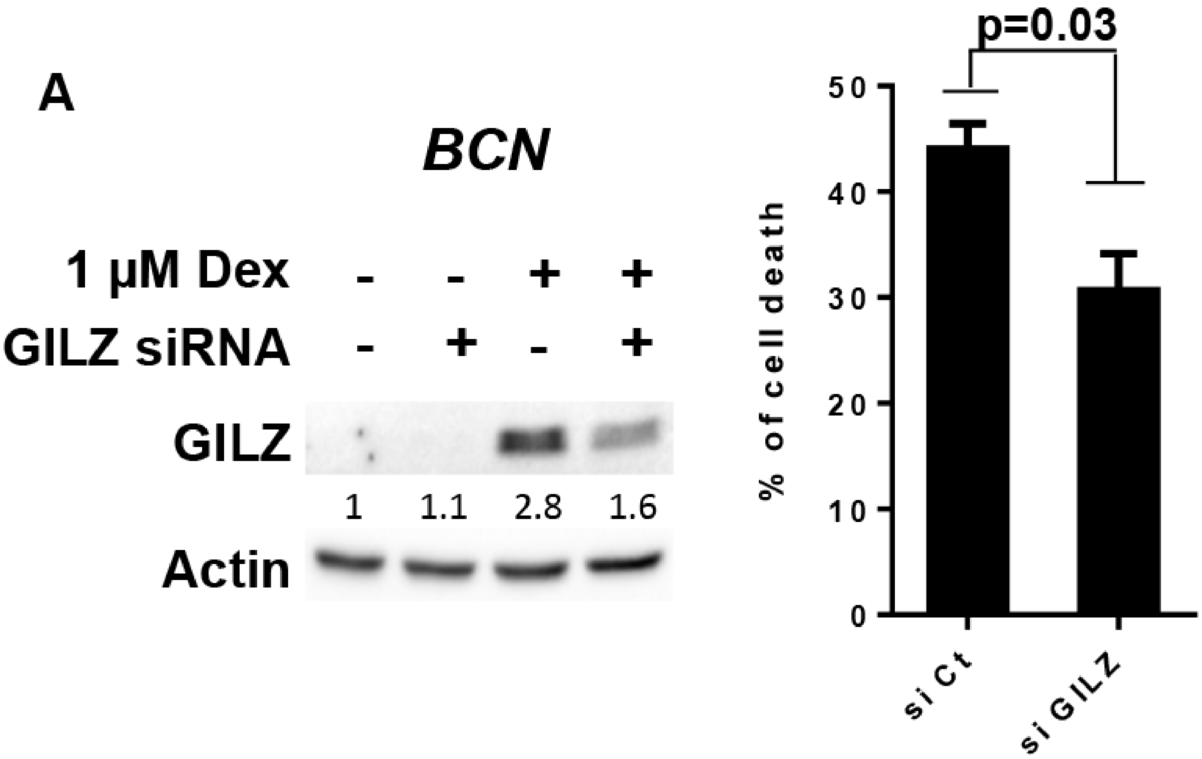

Supplementary Figure S3: A. Following GILZ silencing, BCN cells were treated with Dex. Cell death was assessed by Apo2.7 staining after 48 hours of Dex treatment. Data represent the mean ± SD of 5 independent experiments. Statistical analysis was performed using Wilcoxon matched-pairs signed rank test. After 16 hours of Dex treatment, GILZ silencing was analyzed by immunoblotting.

Supplementary Table S1: Characteristics of the HMCL cohort

HMCL name	Translocation	Molecular subgroup	% cell death	Dex Sensitivity
BCN	t(14;16)	MF	60 ± 8	Sensitive
MM.1S	t(14;16)	MF	47 ± 2	Sensitive
ANBL6	t(14;16)	MF	32 ± 6	Sensitive
L363	t(20;22)	MF	29 ± 3	Sensitive
RPMI8226	t(14;16)	MF	25 ± 2	Sensitive
NAN6	t(14;20)	MF	15 ± 3	Sensitive
JJN3	t(14;16)	MF	8 ± 3	Resistant
NAN1	t(14;16)	MF	7 ± 4	Resistant
XG6	t(16;22)	MF	3 ± 2	Resistant
MM.1SDR	t(14;16)	MF	0 ± 0	Resistant
OPM2	t(4.14)	MS	68 ± 4	Sensitive
NAN8	t(4;14)	MS	62 ± 3	Sensitive
JIM3	t(4;14)	MS	24 ± 5	Sensitive
KMS11	t(4;14)	MS	11 ± 1	Resistant
NCI-H929	t(4;14)	MS	5 ± 4	Resistant
LP1	t(4;14)	MS	1 ± 1	Resistant
NAN8DR	t(4;14)	MS	0 ± 0	Resistant
XG7	t(4;14)	MS	0 ± 0	Resistant
SKMM2	t(11;14)	CCND1	9 ± 1	Resistant
Karpas620	t(11;14)	CCND1	6 ± 4	Resistant
U266	t(11;14)	CCND1	6 ± 2	Resistant
NAN7	t(11;14)	CCND1	0 ± 2	Resistant
XG1	t(11;14)	CCND1	0 ± 1	Resistant
KMS12-BM	t(11;14)	CCND1	0 ± 0	Resistant
KMS12-PE	t(11;14)	CCND1	0 ± 0	Resistant
MDN	t(11;14)	CCND1	0 ± 0	Resistant
NAN10	t(11;14)	CCND1	0 ± 0	Resistant
XG5	t(11;14)	CCND1	0 ± 0	Resistant
XG3	t(14;?)	Others	4 ± 4	Resistant
XG2	t(12;14)	Others	1 ± 4	Resistant
SBN	t(14;?)	Others	1 ± 1	Resistant
KMM1	t(6;14)	Others	0 ± 0	Resistant
AMO1	t(12;14)	Others	0 ± 0	Resistant

The MM cell lines (n = 31) and the two resistant-induced cell lines were screened for the effect induced by Dex 1 µM for 72 hours. Cell death was assessed by Apo2.7 staining. Dex sensitivity was defined as follow: sensitive, ≥ to 15% apoptotic HMCL cells.
